# Do insecure adult attachment styles mediate the relationship between childhood maltreatment and violent behavior?

**DOI:** 10.1017/S0954579422001468

**Published:** 2023-01-26

**Authors:** Nina Papalia, Cathy Spatz Widom

**Affiliations:** 1 Centre for Forensic Behavioural Science, Swinburne University of Technology and Victorian Institute of Forensic Mental Health, Melbourne, VIC, Australia; 2 Psychology Department, John Jay College, New York City, NY, USA; 3 The Graduate Center, City University of New York, New York City, NY, USA

**Keywords:** adult attachment styles, child maltreatment, neglect, physical abuse, prospective longitudinal, violence, violent offending

## Abstract

Attachment theory has played an important role in attempts to understand the “cycle of violence,” where maltreated children are at increased risk for perpetrating violence later in life. However, little is known empirically about whether adult attachment insecurity in close relationships may partly explain the link between childhood maltreatment and violent behavior. This study aimed to address this gap using data from a prospective longitudinal study of documented childhood abuse and neglect cases and demographically matched controls (ages 0–11 years), who were followed into adulthood and interviewed (*N* = 892). Participants completed the Relationship Scales Questionnaire assessing adult attachment styles at mean age 39.54. Criminal arrest data were used to determine arrests for violence after the assessment of attachment through mean age 50.54. There were significant direct paths from childhood maltreatment and adult attachment insecurity to violent arrests after attachment measurement. Attachment insecurity partly explained the higher levels of violence in individuals with maltreatment histories. Analyses of maltreatment subtypes and attachment styles revealed that attachment anxiety appeared to mediate paths between neglect and physical abuse and later violence. There were no significant indirect paths from neglect or physical abuse to violence via attachment avoidance. Implications and future directions are discussed.

## Introduction

Childhood maltreatment is linked to a cascade of negative health, social, psychological, and behavioral outcomes that undermine healthy development over the lifespan (Bunting et al., 2018; Carr et al., 2020; Font & Kennedy, 2022; Jaffee, 2017). Among these, the “cycle of violence” hypothesis originally predicted that physically abused children were at increased risk of committing acts of violence as they mature (Maxfield & Widom, 1996; Widom, 1989b). A large body of research now supports associations between childhood abuse and neglect and violent behavior (Li et al., 2020; Maas et al., 2008), including links with earlier onset and more persistent violence (Herrenkohl et al., 2022; Papalia et al., 2022). Although research relies heavily on cross-sectional designs and retrospective recall of maltreatment, mounting prospective and longitudinal evidence confirms that, while not inevitable, abused and neglected children are at increased risk to engage in violence (Fitton et al., 2020; Widom, 2017). This pattern, which may extend deep into adulthood (Schuck & Widom, 2021), can contribute to an intergenerational cycle. Such findings underscore the importance of identifying mechanisms through which exposure to childhood maltreatment could translate into violent behavior, to help remediate its effects. Although these pathways are complex and multiply determined, attachment theory has played an important role in attempts to understand the cycle of violence. However, few studies have adequately tested its central hypothesis; namely, children who grow up in maltreating environments are vulnerable to insecure attachments, which are relatively stable over the life cycle and can contribute to aggression and violence. This is the focus of the current study.

### Childhood maltreatment and adult attachment styles

According to Bowlby’s (1973) attachment theory, early caregiving experiences shape children’s expectations and beliefs about themselves and the responsiveness and trustworthiness of significant others (Fraley, 2002). These knowledge structures, or “internal working models,” are theorized to lay a foundation for later individual differences in personality (Bowlby, 1973; Sroufe, 2005). A child who receives emotionally nurturing, responsive and reliable caregiving that meets their physical and psychological needs develops internal working models that others will be available and supportive when called upon; that is, they develop secure attachment representations (Ainsworth et al., 1978). Conversely, a child exposed to insensitive, harsh, or unpredictable caregiving develops beliefs that others’ responses to their signals of distress are unpleasant or untrustworthy, creating insecure attachment representations.

Attachment relationships are theorized to be important over the life course. Building on early classifications of infant attachment behavior toward caregivers (Ainsworth et al., 1978; Main & Solomon, 1986), social psychology researchers have sought to characterize individual differences in the beliefs and insecurities adults hold about themselves and their relationships with close others (Bartholomew & Horowitz, 1991; Fraley & Roisman, 2019; Hazan & Shaver, 1987). Empirical work suggests that adult attachment styles are distributed across two dimensions – avoidance and anxiety (Brennan et al., 1998). Adults who are relatively avoidant are uncomfortable opening up to others, may inhibit and control their emotions, and preference self-sufficiency over close relationships. Relatively anxious people worry that others will not be available and responsive in times of need and have difficulty regulating intense negative emotions. Longitudinal research finds that adult attachment styles have their origins, in part, in early caregiving experiences (Fraley et al., 2013; Fraley, 2002) and are linked to psychopathology (Fairbairn et al., 2018; Widom et al., 2018).

Childhood maltreatment, encompassing abuse or neglect, is considered an important cause of insecure parent–child attachments (Baer & Martinez, 2006; Carr et al., 2020; Cyr et al., 2010; Vasileva & Petermann, 2018), however its power in forecasting adult attachment styles in other close relationships is less clear. Some research supports a cross-sectional link between abusive and neglectful childhood environments and insecure attachment styles in adulthood (Crow et al., 2021; Hocking et al., 2016; Kim et al., 2021; Zietlow et al., 2017). A handful of longitudinal studies also suggest that child maltreatment may have enduring consequences for adults’ self-reported attachment styles (Dion et al., 2019; Widom et al., 2018) and their interview-elicited attachment representations of romantic partners (Raby et al., 2017).

Attachment theory recognizes that different types of maltreatment by caregivers may affect children’s internal working models in different ways. Over time, these models are believed to form a template for adults’ working models of other close relationships. Neglect, for example, represents deprivation of basic wellbeing and safety needs and may be experienced as abandonment and rejection (Gauthier et al., 1996). Neglected children may learn they are ineffective in communicating their needs and obtaining support, and so intensify their demands for attention and care, or become helpless in the absence of a successful strategy (Crittenden & Ainsworth, 1989; Cyr et al., 2010). They may construct schemas of themselves as unworthy and unable to cope independently (Pilkington et al., 2021) and develop intense concern that others will reject or abandon them (i.e., attachment anxiety). Physical abuse, on the other hand, involves threat of harm to the child’s physical integrity and may lead to working models of the world as threatening and dangerous. Physically abused children may become hypervigilant toward hostile cues, mask their feelings to avoid upsetting caregivers, and learn that it is safer to be self-reliant. This may contribute to distrust of others and avoidance in close relationships for individuals with histories of physical abuse.

Research on these conceptual links between childhood neglect, physical abuse, and adult attachment styles is mixed. A meta-analysis of cross-sectional studies found that both maltreatment types were related to both avoidant and anxious attachment styles (Kim et al., 2021). Therefore, neglectful experiences may also lead to adults’ avoidance through beliefs that others cannot be relied on for support, and physical abuse may be linked to an anxious style if others are seen as harmful and rejecting. Recent prospective longitudinal work gives a more complicated picture. Among 116 individuals followed to mean age 25 years, Raby et al. (2017) found that neither neglect nor physical abuse uniquely predicted participants’ dismissing or pre-occupied attachment representations in adult romantic relationships as elicited by the Current Relationship Interview (CRI). However, exposure to chronic or multiple-type maltreatment increased risk for dismissing CRI representations. A larger study (*N* = 650) found that both neglect and physical abuse were associated with self-reported anxious attachment styles in close relationships at mean age 39 years, whereas only neglect related to avoidant styles (Widom et al., 2018). These findings underscore the importance of further probing the relationships between different experiences of abuse and neglect and adult attachment insecurities.

### Adult attachment styles and violence

Separate literatures have examined the relationship between attachment and violent behavior across the life span. Insecure child–parent attachments have been linked to early aggression (Madigan et al., 2016; Savage, 2014), and some work has found associations between insecure attachment styles in other close relationships and violent and sexual offending (Grady et al., 2021; Ogilvie et al., 2014; Yoder et al., 2020), intimate partner violence (Dutton & White, 2012; Velotti et al., 2022), and child maltreatment perpetration/potential (Lo et al., 2019). Although the potential mechanisms of these relationships are poorly understood, adult attachment insecurity is associated with problems in functioning (e.g., personality pathology, maladaptive emotion regulation, dysfunctional social relations, poor coping with stress) that, in turn, may increase risk of violence in different contexts (Crawford et al., 2006; Levy et al., 2015; Mikulincer & Shaver, 2016).

Theoretically, both adult attachment insecurities (avoidance and anxiety) could influence violence risk, but the pathways may differ (see Mauricio et al., 2007). Drawing on the partner violence literature, individuals higher in attachment anxiety are hyper-sensitive to relational threat and may appraise ambiguous partner behavior and conflict in catastrophic terms (Mikulincer & Shaver, 2016). Faced with attachment-related fears, they may display uncontained anger and rumination that interferes with effective communication (Senkans et al., 2020). Partner violence driven by an anxious attachment style may be motivated by a desire to (re)gain proximity to the attachment figure and prevent abandonment (Bartholomew & Allison, 2006). Attachment anxious individuals’ tendency toward intense negative affect, underregulation of emotions, and impulsivity could also contribute to aggression across contexts (Levy et al., 2015; Roberton et al., 2012).

Conversely, attachment avoidant individuals view real or perceived bids for intimacy and vulnerability as aversive (Senkans et al., 2020). They may minimize significant others’ feelings and distance themselves emotionally and physically (Mikulincer & Shaver, 2016). When circumstances do not allow withdrawal, avoidant individuals may use aggression to dominate others and preserve self-sufficiency. Their tendencies for defensive self-enhancement, suppressed anger/hostility, and impaired empathy also represent potential pathways to violence (Bartholomew & Allison, 2006; Mikulincer & Shaver, 2016; Mikulincer, 1998; Roberton et al., 2012). A body of work – largely with offending males, clinical samples, or partner abusers – supports these associations between the two insecure adult attachment styles and violent/aggressive outcomes, with effects sizes generally larger for anxiety (Critchfield et al., 2008; Dutton & White, 2012; Fossati et al., 2009; Mauricio et al., 2007; Ogilvie et al., 2014; Velotti et al., 2022).

Recognizing the links between adult attachment styles and violence, adult attachment insecurity may represent a pathway to violent behavior for those who have experienced maltreatment (Dutton & White, 2012; Grady et al., 2017; Widom & Wilson, 2015). Some literature supports the role of attachment as a potential mediator of associations between child physical abuse and aggressive behavior in childhood and adolescence (Finzi et al., 2001; Herrenkohl et al., 2003). However, few studies have examined whether these relationships extend to attachment styles in close adult relationships. Drawing again from the intimate partner abuse literature, some evidence suggests that romantic attachment insecurities (particularly an anxious style) may contribute to the increased risk of partner violence seen in adults who recall exposure to parental violence in childhood (Godbout et al., 2017; Lee et al., 2014). Other work focusing on general antisocial attitudes and behaviors found that adult attachment avoidance mediated pathways from childhood abuse and adversity (Oshri et al., 2015; Yang & Perkins, 2021).

### Summary of gaps

There are important limitations and knowledge gaps in the extant literatures on childhood maltreatment, adult attachment insecurity, and violence. First, most studies provide a cross-sectional snapshot of associations, making it challenging to disentangle temporal order (for reviews, see Kim et al., 2021; Ogilvie et al., 2014). Where longitudinal designs are employed, these are relatively short term and rarely extend past early adulthood (Dion et al., 2019; Godbout et al., 2017). Second, the few studies that explore adult attachment styles as mediators in the cycle of violence focus on intimate partner abuse (Godbout et al., 2017; Lee et al., 2014). Yet, empirical work supports associations between adult attachment insecurity and a wider range of violent outcomes (Ogilvie et al., 2014). Third, there is a near absence of research examining adult attachment insecurity’s role in the childhood neglect–violence relationship. This is despite neglect being the most prevalent form of reported maltreatment in the United States (U.S. Department of Health & Human Services, 2022) and compelling evidence linking it to violent offending in adulthood (Maxfield & Widom, 1996; Schuck & Widom, 2021). We are aware of no published study that has longitudinally examined whether adult attachment insecurity in close relationships mediates the associations between objectively determined child abuse and neglect and subsequent violent behavior.

### Current study and statement of hypotheses

Using data from a prospective longitudinal study of childhood maltreatment’s consequences, this research aims to examine if insecure adult attachment styles play a role in explaining the cycle of violence. Adult attachment styles were assessed in middle adulthood. Here, to maintain correct temporal sequencing, we examine violence (using criminal arrests) after attachment was assessed. This approach is conservative because participants were mean age 39 at the time of attachment assessment and past the peak years for violent offending. We focus our hypotheses on the expected paths from any maltreatment and from physical abuse and neglect specifically. We do not test hypotheses about possible paths from sexual abuse to violent arrests via adult attachment insecurity. This is because earlier published work with this dataset found limited support for an association between child sexual abuse and violent arrests, controlling for age, race, and sex (Maxfield & Widom, 1996).

Informed by prior literature, we have five key hypotheses. First, individuals with histories of childhood maltreatment will show greater adult attachment insecurity than non-maltreated controls (H1). Second, although we expect each type of maltreatment to relate to each attachment style, we hypothesis that neglect might be more strongly associated with attachment anxiety, whereas physical abuse might be more strongly linked to attachment avoidance (H2). Third, adult attachment insecurities will predict an increased likelihood of subsequent arrests for violence (H3). Fourth, adult attachment insecurity will be a mechanism linking child maltreatment to later violence (H4). More specifically, adult attachment anxiety will partly mediate the neglect–violence relationship and adult attachment avoidance will partly mediate the physical abuse–violence relationship (H5).

## Method

### Design and participants

The description of the methods in this study are similar to those previously published (Widom, 1989a). Briefly, the data were collected as part of a large, prospective cohort study, in which abused and/or neglected children and demographically matched non-victimized children were followed prospectively into adulthood. Because of the matching procedure, the children are assumed to differ only in the risk factor; that is, having experienced childhood physical or sexual abuse or neglect. The assumption of equivalency for the groups is an approximation, since random assignment was not possible.

The original sample of maltreated children (*N* = 908) represented all substantiated cases of childhood physical and sexual abuse and neglect processed in the county juvenile (family) and adult criminal courts of a Midwestern metropolitan area from 1967 through 1971. Maltreatment cases were restricted to children aged 0–11 years at the time of the incident. *Physical abuse* cases included injuries such as bruises, welts, burns, abrasions, lacerations, wounds, cuts, bone and skull fractures, and other evidence of physical injury to the child. *Sexual abuse* cases included felony sexual assault, fondling or touching, rape, sodomy, and incest. *Neglect* cases reflected a judgment that the parents’ deficiencies in caregiving were beyond those found acceptable by community and professional standards at the time and represented extreme failure to provide adequate food, clothing, shelter, and medical attention to children. Excluded from this sample of neglect cases were those that represented: (a) adoption of the child as an infant; (b) “involuntary” neglect only – usually resulting from the temporary institutionalization of the legal guardian; (c) placement only; or (d) failure to pay child support.

A critical element of the design involved selecting a matched control group. Controls were matched with the maltreatment sample based on age, sex, race/ethnicity, and approximate family social class during the time period under study. The matching procedure for the latter used a broad definition of social class that included neighborhoods in which children were reared and schools they attended. Any control child with an official record of abuse or neglect was excluded, regardless of whether the record was before or after the period of the study.

Using county birth record information, children under school age at the time of the abuse and/or neglect were matched with children of the same sex, race, date of birth (± 1 week), and hospital of birth. Children of school age were matched as closely as possible by sex, race, date of birth (± 6 months), and class in the elementary school system during 1967 through 1971. Records of more than 100 elementary schools for the same time period were used to find matches. Overall, there were matches for 73% of abused and neglected children, resulting in 667 control children in the original sample.

In the first phase of the larger study, maltreated children and controls (total *N* = 1,575) were followed up through an examination of official criminal records. Later phases involved locating and interviewing both groups at different time periods. The current sample included 892 of the 896 participants who took part in the second interview, when adult attachment was assessed (four participants were excluded due to invalid interview data). Despite attrition associated with death, refusals, and an inability to locate individuals over the various waves of the study, the characteristics of the groups across the initial three phases of the study have remained about the same (see Table 1). Bivariate analyses of key characteristics between participants in the second interview versus those who did not complete the second interview showed no significant differences in child maltreatment status, race, and age. Females were more likely to participate in the second interview than males (OR = 1.43, 95% CI: 1.10–1.85). Together, these analyses indicate that selective attrition is not a significant problem in our study.

The present sample included 497 (55.7%) individuals with documented histories of childhood maltreatment and 395 (44.3%) controls. Mean age at interview was 39.52 years (*SD* = 3.53; range 30–47) and approximately half the sample were female (*n* = 457, 51.2%). Regarding race/ethnicity, 60.9% (*n* = 543) self-identified as White, non-Hispanic, 32.5% (*n* = 290) as Black, non-Hispanic, 2.8% (*n* = 25) as Black Hispanic, 1.7% (*n* = 15) as Native American, 1.6% (*n* = 14) as White Hispanic, and a very small number identified as Hawaiian Islanders or with other racial and ethnic groups. There were no significant differences between the abused/neglected group and controls in terms of sex, race/ethnicity, or age.

### Procedures

Participants were interviewed in-person in their homes or other quiet locations of their choice. Interviewers and participants were blind to the purpose of the study and the inclusion of an abused and neglected group. Participants were told that they had been selected to participate as part of a large group of individuals who grew up in that area in the late 1960s and early 1970s. Institutional Review Board approval was obtained for the procedures involved in this study, and individuals who participated signed a consent form acknowledging that they understood the conditions of their participation and were participating voluntarily. For those individuals with limited reading ability, the consent form was read to the person and, if necessary, explained verbally.

### Measures

#### Adult attachment styles

Adult attachment styles were assessed using the Relationship Scales Questionnaire (RSQ; Griffin & Bartholomew, 1994), a self-report measure containing 30 statements. Participants were asked to rate on a 5-point scale how well each statement represented their experiences in “close relationships” (1 = *Not at all like me*, 5 = *Very much like me*); three items referred specifically to “romantic partners.” The RSQ was developed as an omnibus tool that includes items from several commonly used adult attachment style measures, allowing researchers to derive attachment styles using different conceptualizations. However, existing evidence supports the use of two attachment dimensions: attachment-related avoidance (e.g., discomfort with intimacy and relying on significant others) and attachment-related anxiety (e.g., worry about the availability and responsiveness of significant others) (Brennan et al., 1998). Prior confirmatory factor analyses of the RSQ comparing common measurement models suggested Simpson et al.’s (1992) 13-item, two-dimensional model provided the best fit to the data (Roisman et al., 2007). As such, we used this operationalization of attachment avoidance (eight items, e.g., “I find it difficult to trust others completely”) and attachment anxiety (five items, e.g., “I often worry that romantic partners don’t really love me”) in our study. Roisman and colleagues found that the two dimensions showed good internal consistency and correlated strongly with internalizing and externalizing psychopathology under conditions of low and high life stress (Fortuna & Roisman, 2008; Roisman et al., 2007). In the current study, Cronbach’s alpha values were α = .68 for avoidance and α = .80 for anxiety. Mean item response scores for the two scales were significantly correlated (*M*_Avoid_ = 2.75, *SD* = 0.74; *M*_Anxiety_ = 2.16, *SD* = 0.99; *r* = .49, *p* < .001).

#### Official arrests for violence

Whether a participant had been arrested for a violent crime was determined from criminal history checks conducted during 1987–1988, 1994, and 2013 (Maxfield & Widom, 1996; Widom & Massey, 2015; Widom, 1989b). The updated arrest record searches included information from both the Federal Bureau of Investigation’s (FBI) National Crime Information Center (NCIC) and state law enforcement records for the Midwestern state in which childhood maltreatment cases were initially ascertained. The information obtained from these three searches was combined to create a lifetime criminal history record for each participant through mean age 50.54 years (*SD* = 3.49). Arrests for violent crimes included murder and attempted murder, manslaughter and involuntary manslaughter, reckless homicide, rape, sodomy, robbery and robbery with injury, assault, assault and battery, aggravated assault, and battery and battery with injury. We examined the presence of any arrests for violence after the adult attachment assessment (0 = no [*n* = 798, 89.5%] and 1 = yes [*n* = 94, 10.5%]). Because most interviews were completed in 2000, this time was selected as the year from which to examine whether adult attachment predicted subsequent arrests for violence through 2013. We used a binary rather than count variable for violent arrests because we expected violent offending to be relatively infrequent during middle adulthood.

#### Control variables

All models controlled for sex, age, and race/ethnicity. Sex was coded as 0 = male and 1 = female. Participant race/ethnicity were determined by showing participants a card with the names of racial and ethnic groups and having them indicate which best described them. Race was coded as 1 = White, non-Hispanic and 0 = all other groups. Age (in years) at the time of adult attachment assessment was included as a covariate.

### Statistical Analyses

Descriptive analyses were undertaken in R (R Core Team, 2021) and structural equation models (SEMs) were developed in MPlus (Muthén & Muthén, 1998–2017). The first step was to examine zero-order bivariate relationships between observed study variables using Pearson correlations. Next, we conducted confirmatory factor analysis (CFA) to determine whether the eight attachment avoidance items and five attachment anxiety items to be included in the SEMs represented latent constructs with acceptable psychometric properties. Preliminary CFA showed that all three of the negatively worded items loaded relatively weakly (<.20) onto their hypothesized factor (i.e., the avoidance dimension, in all three cases), suggesting the likely presence of a systematic method effect (Ye & Wallace, 2013). We dealt with this by allowing the error terms of the negatively worded items to correlate rather than specifying a distinct method factor. In the final CFA, factor loadings for attachment avoidance were 0.10 (*p* = .007), 0.08 (*p* = .021), and 0.09 (*p* = .013) for the three negatively worded items and ranged from 0.50 to 0.78 (*p* < .001) for positively worded items. For attachment anxiety, factor loadings ranged from 0.51 to 0.77 (*p* < .001). The two latent dimensions were significantly correlated (*r* = 0.75, *p* < .001). The statistically significant factor loadings, together with acceptable model fit indices, suggest the two latent attachment constructs are adequate and can be used in subsequent SEMs.

The final step involved three probit SEMs to test for mediation. First, we tested a model with adult attachment insecurity as the latent mediator (with mean item response scores for the two insecurity dimensions as indicator variables), any childhood maltreatment as the independent variable, and any arrest for violence after 2000 as the dependent variable. We then tested two separate models with each latent attachment dimension (avoidance and anxiety) as the mediator, with childhood neglect and physical abuse entered as dummy coded independent variables (controls were the reference category). All SEMs used the Weighted Least Squares estimation with Means and Variances adjustment (WLSMV) and the default Delta parametrization. We report standardized probit regression coefficients and 95% confidence intervals, which represent the difference in *z*-score associated with a one-unit change in the independent variable. A statistically significant coefficient is identified by confidence intervals that do not overlap with zero. Several indices were considered in assessing overall model fit (i.e., root mean square error of approximation [RMSEA], comparative fit index [CFI] and Tucker–Lewis index [TLI]), as were individual path estimates and indirect effects, their size, and statistical significance. We also report *R*^2^ values as a measure of effect size. All SEMs controlled for sex, age, and race/ethnicity.

## Results

### Descriptive analyses

Table 2 presents the descriptive statistics for all observed variables included in the study, separately for maltreated individuals and controls. The bivariate zero-order correlations between all variables are shown in Table 3. Any childhood maltreatment, and neglect and physical abuse specifically, were positively associated with adult attachment avoidance and anxiety. Maltreated individuals were also significantly more likely to be arrested for a violent crime after attachment assessment compared to controls. Attachment avoidance and anxiety correlated positively with subsequent arrests for violent crimes.

### Child maltreatment, adult attachment insecurities, and arrests for violence

**Figure 1.**
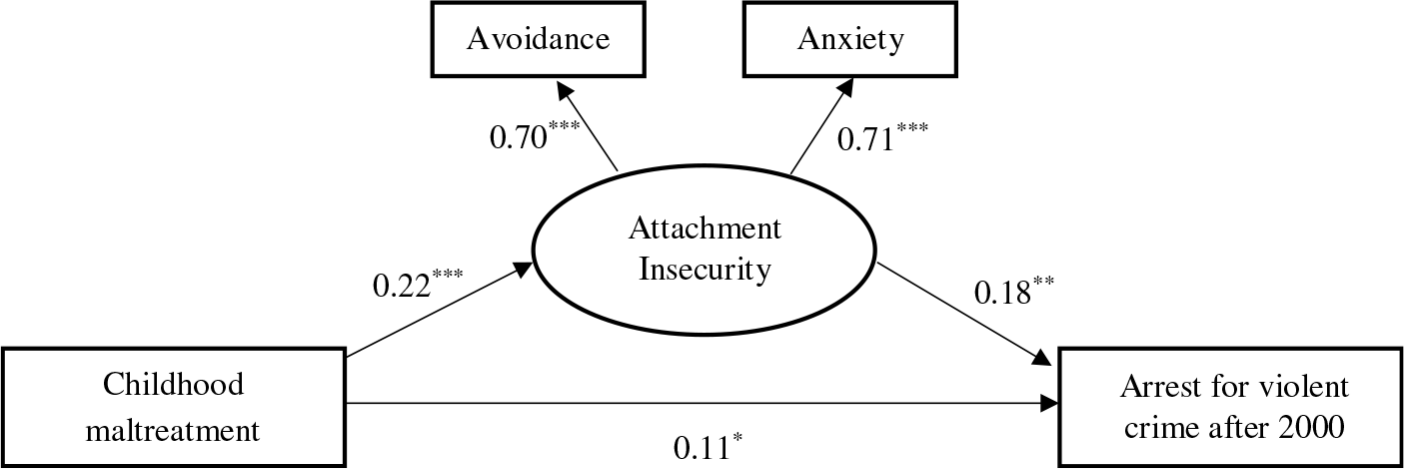
Structural equation model showing paths from maltreatment to arrests for violence through adult attachment insecurity. *Note.* Standardized betas are presented. All lines are solid, reflecting significant paths. Analyses control for age, sex, and race. Model fit indices: RMSEA = 0.02, CFI/TLI = 0.99/0.97. *R*^2^ = 0.05 (attachment insecurity) and 0.22 (arrests for violence). *p < .05, * *p < .01, ***p < .001.

An SEM was run to assess links between any childhood maltreatment, adult attachment insecurity, and subsequent arrests for violence (see Figure 1). Examination of the fit statistics indicated that this model provided an acceptable fit to the data (RMSEA = 0.02, CFI = 0.99, TLI = 0.97). The mean item response scores for both avoidance (β = 0.70, 95% CI [0.57, 0.83], *p* < .001) and anxiety (β = 0.71, 95% CI [0.57, 0.85], *p* < .001) dimensions loaded strongly onto the latent attachment insecurity variable. Childhood maltreatment positively predicted adult attachment insecurity (β = 0.22, 95% CI [0.14, 0.30], *p* < .001), which in turn predicted an increased likelihood of a subsequent arrest for violence (β = 0.18, 95% CI [0.05, 0.31], *p* = .007). The indirect path from childhood maltreatment to violent arrests after 2000 via attachment insecurity was significant (β = 0.04, 95% CI [0.01, 0.07], *p* = .015), meaning adult attachment insecurity partly mediated the relationship between maltreatment and later violence. It is noteworthy that the direct path from childhood maltreatment to arrests for violence after 2000 remained significant, controlling for sex, age, and race (β = 0.11, 95% CI [0.004, 0.22], *p* = .043). The *R* square value for the prediction of arrests for violence was 0.22 (*p* < .001), indicating a large effect.

**Figure 2.**
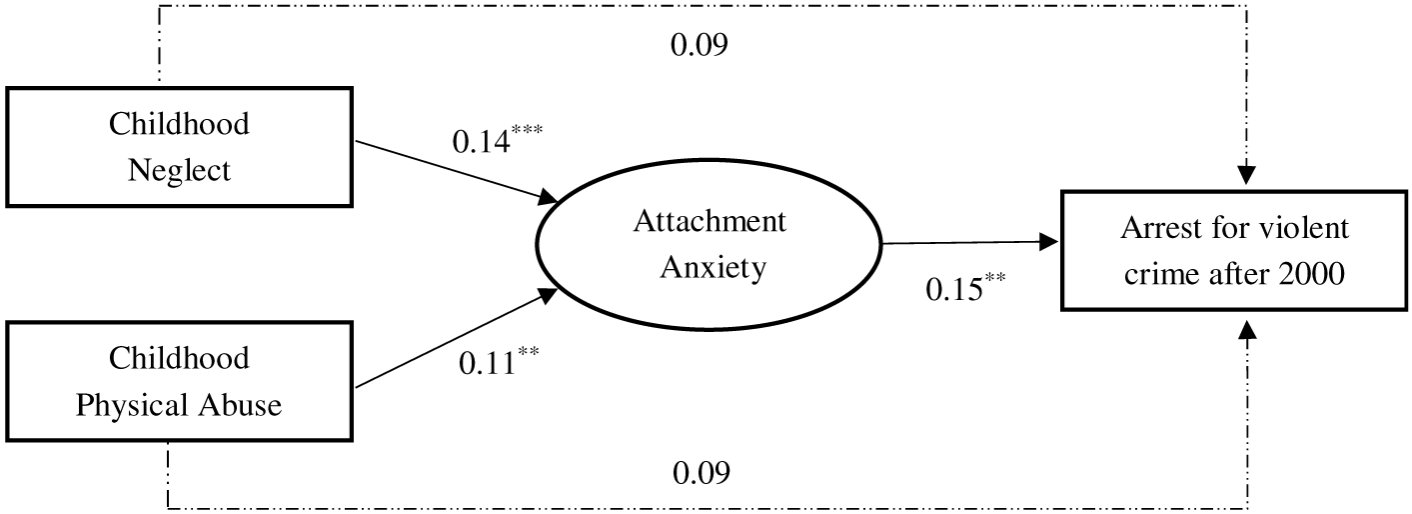
Structural equation model showing paths from childhood neglect and physical abuse to arrests for violence through adult attachment anxiety. *Note.* Standardized betas are presented. Solid lines are significant paths, dotted lines are not significant. Analyses control for age, sex, and race. Model fit indices: RMSEA = 0.05, CFI/TLI = 0.89/0.88. *R*^2^ = 0.03 (attachment anxiety) and 0.21 (arrests for violence). *p < .05, **p < .01, ***p < .001.

**Figure 3.**
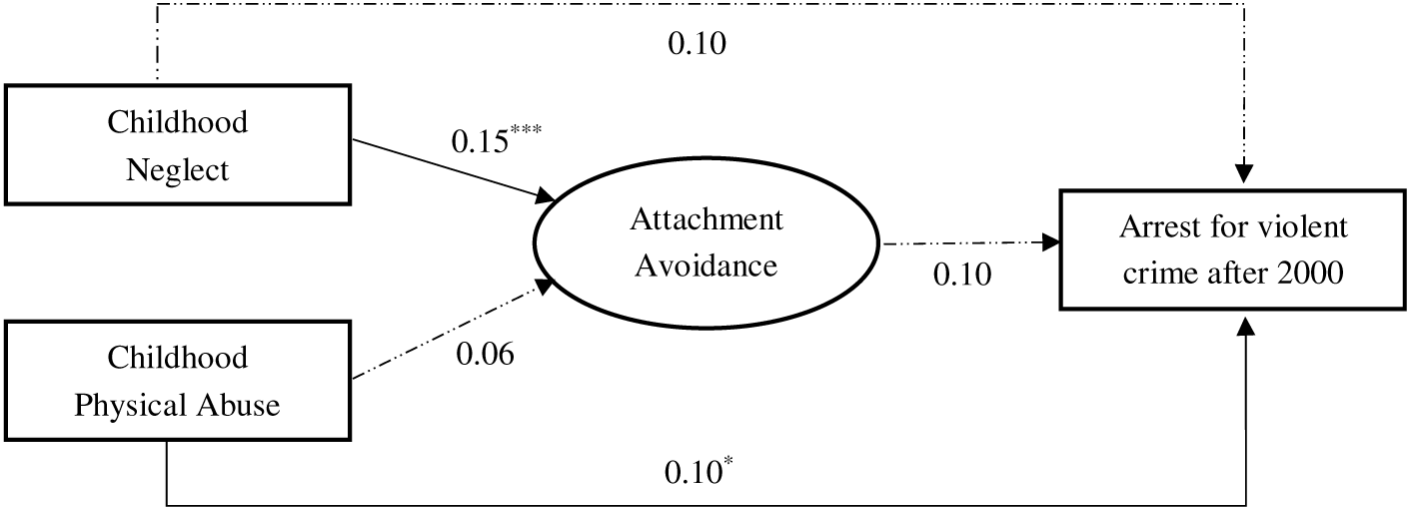
Structural equation model showing paths from childhood neglect and physical abuse to arrests for violence through adult attachment avoidance. *Note.* Standardized betas are presented. Solid lines are significant paths, dotted lines are not significant. Analyses control for age, sex, and race. Model fit indices: RMSEA = 0.05, CFI/TLI = 0.86/0.80. *R*^2^ = 0.03 (attachment avoidance) and 0.20 (arrests for violence). *p < .05, **p < .01, ***p < .001.

The results for the two models examining paths from childhood maltreatment subtypes (neglect and physical abuse) to arrests for violence via attachment anxiety and avoidance are shown in Tables 4 and 5, respectively. We also present the models in Figures 2 and 3 – for anxiety and avoidance, respectively – for ease of interpretation. As shown, RMSEA values were 0.05 for both models suggesting acceptable model fit, and CFI and TLI values approached 0.90, a common threshold for acceptable fit (Hu & Bentler, 1998). Inspection of path coefficients suggested that childhood neglect comparably predicted both attachment anxiety (β = 0.14, *p* < .001) and avoidance (β = 0.15, *p* < .001), whereas physical abuse was significantly related to attachment anxiety (β = 0.11, *p* = .003) but not avoidance (β = 0.06, *p* = .082). Adult attachment anxiety predicted future arrests for violent crimes (β = 0.15, *p* = .008) but the path from attachment avoidance to violence was not statistically significant (β = 0.10, *p* = .071).

In the attachment anxiety model (Figure 2), the two indirect paths from childhood neglect (β = 0.02, *p* = .029) and childhood physical abuse (β = 0.02, *p* = .048) were statistically significant. That is, there was evidence that attachment anxiety may mediate the relationships between both neglect and physical abuse and later arrests for violent crimes; however, confidence intervals for the indirect effects were near to zero (Table 4). Neither of the maltreatment subtypes had a significant direct effect on later violence in this model. The *R* square value for the prediction of violent arrests was 0.21 (*p* < .001), suggesting a large effect.

In the attachment avoidance model (Figure 3), the indirect paths from childhood neglect (β = 0.02, *p* = .099) and childhood physical abuse (β = 0.01, *p* = .21) were not statistically significant. That is, adult attachment avoidance did not significantly mediate the relationships between maltreatment subtypes and violent arrests after 2000. In this model, childhood physical abuse directly predicted subsequent violent arrests (β = 0.10, *p* = .034), whereas neglect did not (β = 0.10, *p* = .087). Overall, the *R* square value (0.20, *p* < .001) suggested a large effect for the prediction of arrests for violence after 2000.

## Discussion

This was the first study to longitudinally examine indirect paths from objectively determined childhood abuse and neglect to violent arrests through adult attachment styles. We found that general adult attachment insecurity at mean age 39 predicted an increased likelihood of future arrests for violence through mean age 50. Attachment insecurity, in part, explained the heightened propensity for violence in individuals with histories of childhood maltreatment. Further analyses of maltreatment subtypes and insecurity dimensions revealed that adult attachment avoidance did not significantly mediate relationships between childhood neglect or physical abuse and later violent arrests. Conversely, there was some evidence that adult attachment anxiety may play a mediating role in the links between both maltreatment types and violence.

Our findings support earlier prospective evidence that the impacts of childhood abuse and neglect on attachment insecurity may extend to adult relationships (Raby et al., 2017; Widom et al., 2018). Supporting our first hypothesis, maltreated individuals showed greater attachment insecurity approximately 30 years after the abuse/neglect occurred, compared to non-maltreated individuals. This association was independent of age, sex, and race. Hypothesis two was partially supported, where childhood neglect predicted adult attachment anxiety as expected, as did childhood physical abuse. In contrast to our prediction, we did not find (in multivariable models) that adults with histories of childhood physical abuse had greater levels of attachment avoidance than controls. This may be partly influenced by reduced power to detect relatively smaller path estimates for the physical abuse subsample. Neglected individuals, however, were significantly more attachment avoidant than non-maltreated individuals. Thus, in addition to their tendency toward excessive worry about being abandoned, rejected and unworthy, neglected individuals in our sample showed defensive, mistrustful, and avoidant orientations in close relationships, perhaps to protect against rejection (see Kim et al., 2021).

These findings relating to maltreatment subtypes broadly replicate earlier work involving a smaller sample from this dataset (Widom et al., 2018) but diverge from Raby et al.’s (2017) prospective study. Raby et al., found that abuse/neglect subtypes did not predict adults’ romantic attachment representations (pre-occupied or dismissive), however more chronic and multiple-type maltreatment were linked with risk of dismissive representations. We did not have data on the chronicity of maltreatment in our sample, and relatively few maltreatment cases (11%) had more than one type of documented abuse/neglect. Other differences between the current study and Raby et al., with respect to sample size (892 vs. 116), age (mean 39 vs. 25 years) and method of attachment assessment (self-reported attachment styles in close relationships vs. CRI interview protocol for current romantic partnerships) may explain the divergent findings.

Our third hypothesis that adult attachment insecurity would predict an increased likelihood of subsequent arrests for violence was supported. Higher general attachment insecurity was associated with violent arrests, controlling for age, sex, race, and maltreatment status. Said another way, adults who were relatively secure in their close relationships were less likely to have a future arrest for violence, suggesting a possible protective effect of attachment security. These findings support prior work showing that individuals with violent/sexual offenses and family violence behavior (Lo et al., 2019; Ogilvie et al., 2014; Velotti et al., 2022) are relatively more insecure, and extend this work by demonstrating adult attachment’s predictive significance for future violent arrests.

Although simple bivariate analyses showed associations between both attachment style dimensions and later violent arrests, only attachment anxiety predicted violence in SEMs. That is, adults who were relatively anxious were at increased risk of being arrested for violence but the same was not true of relatively avoidant individuals. This contrasts with earlier cross-sectional work linking attachment avoidance with violent/aggressive (Ogilvie et al., 2014; Velotti et al., 2022) and other antisocial outcomes (Oshri et al., 2015; Yang & Perkins, 2021). One possible explanation is that avoidance may be associated with specific types of violence (e.g., intimate partner abuse), or related to violence under certain conditions, which is masked by the present analysis. For example, attachment avoidance may be related to violent behavior only in individuals who are also relatively anxious or who are paired with an attachment anxious partner (Doumas et al., 2008; Velotti et al., 2022). Alternatively, perhaps highly avoidant individuals’ defensive strategies (e.g., compulsive self-reliance, emotional distance and suppression) are so entrenched in this sample that they are socially reclusive, with fewer opportunities for aggression to manifest.

Controlling for age, sex, and race, general attachment insecurity partly explained the association between childhood maltreatment and violent arrests in middle adulthood, providing support for our fourth hypothesis. However, there was partial support for our fifth hypothesis. For both neglected and physically abused children, there were significant indirect paths through attachment anxiety to violence; however, confidence intervals around these indirect paths approached zero suggesting more research is needed to confirm these effects. Furthermore, we did not find evidence that avoidance mediated links between physical abuse (or neglect) and later violent arrests, as we had hypothesized. These findings mirror earlier work suggesting that adult attachment insecurity may have played a mediating role in associations between recalled parental violence in childhood and partner abuse (Godbout et al., 2017; Lee et al., 2014). We advance this knowledge by demonstrating these pathways: (a) exist when examined prospectively over a 40-year period, (b) appear relevant for neglected children and physically abused children, and (c) apply to general violent crimes in middle adulthood.

However, attachment insecurity is relatively common in the general population and prevalent in maltreated individuals (Cyr et al., 2010; van Ijzendoorn, 1995). This contrasts with violent offending, which is relatively infrequent (Ogilvie et al., 2014). Thus, adult attachment insecurity alone cannot explain the cycle of violence, rather it must translate into, or interact with, other individual, social, and contextual risk factors for violence. For example, angry temperament, poor emotion regulation, impulsivity, and disturbed sense of self have been linked to attachment insecurity and may contribute to risk for aggression (Levy et al., 2015; Roberton et al., 2012), especially in maltreated individuals who develop schemas and attitudes that condone the use of violence, are involved with antisocial peers, or experience neighborhood disadvantage and disorder (Herrenkohl et al., 2003; Widom, 2017). There is also some evidence that deficits in mentalizing abilities (i.e., an individual’s capacity to read and understand mental states in others and self) may be a factor underlying the linkages between attachment and violent/aggressive outcomes (Fossati et al., 2009; Levinson & Fonagy, 2004).

Finally, these new results provide further support for the long-range impacts of child maltreatment on violent behavior. The direct path from maltreatment to violent arrests between mean age 39 and 50 remained significant, despite including the indirect pathway via attachment and controls for sex, age, and race. Further work is needed to understand other factors contributing to the cycle of violence during this time in the life course – a period well beyond the peak years of committing violent offenses.

### Limitations

Several caveats to the findings must be noted. We assessed adults’ self-reported attachment-related thoughts and feelings in close adult relationships, not adults’ interview-elicited attachment representations regarding childhood experiences with caregivers. Therefore, we do not know whether participants’ attachment styles are a continuation of their child–caregiver attachments, or the role of other life experiences in shaping adult attachment styles. Attachment (in)security may change over time and relational contexts, and proximal interpersonal experiences and stressors may more powerfully explain adults’ attachment styles than distal ones (Fraley & Roisman, 2019). Future longitudinal work could include repeated assessments with measures from both adult attachment traditions and consider other factors that may influence links between maltreatment and adult attachment. As noted earlier, our violence measure is conservative as (a) we examined arrests over a 13-year period in middle adulthood and (b) many violent acts are not reported to police. Relatedly, we do not know whether attachment insecurity predicts violence *onset* or whether there is a reciprocal relationship between attachment and violence (e.g., violent acts may push others away, reinforcing insecure representations). Cross-lagged panel designs that include self-report and official measures of violence/abuse, broadly defined, may help to address these limitations. The lower internal consistency of the attachment avoidance measure (α = .68) is also a weakness of this study that might have affected estimates of its relation to other variables.

This investigation did not examine whether hypothesized pathways varied by culture or gender, though we controlled for these factors. Future research should consider how the unique stressors and resilience factors experienced across cultural groups may influence attachment and its downstream effects. We also did not have reliable information about who perpetrated the maltreatment or the frequency and chronicity of these experiences, which may impact the association with adult attachment (Raby et al., 2017). Finally, our sample grew up in one Midwestern area in the late 1960s and early 1970s, are skewed toward lower socioeconomic backgrounds, and reflect court-substantiated maltreatment cases. The findings cannot be generalized to children from other regions or time periods, middle- or upper-class families, or whose abuse/neglect was not processed through the courts.

### Implications and conclusions

Recognizing that adult attachment styles seem to originate, in part, from early caregiving experiences, our findings support the importance of early interventions to improve the child–caregiver(s) relationship quality in families with (high risk of) maltreatment. Prior and emerging evidence demonstrates the efficacy of caregiver interventions in fostering child attachment security (Cicchetti et al., 2006; van IJzendoorn et al., 2022), and our findings suggest such interventions may confer additional benefits for reducing long-range violence. Further (longitudinal) study of attachment insecurity’s possible role in the intergenerational transmission of family violence and maltreatment, may help us to understand the broader implications of early child–parent interventions for violence prevention.

The current findings may also have implications for therapeutic interventions for adults with maltreatment histories, particularly those with arrests for violence. Adult attachment, as measured in this study, is conceptualized as a theory of personality development and dynamics (Roisman et al., 2007). Further, meta-analytic work finds relatively modest pooled effects for existing violent offending interventions (Papalia et al., 2019). Therefore, treatments with relatively insecure, violent adults may benefit from incorporating principles from evidence-based personality interventions (e.g., schema-focused therapy, mentalization based therapy) that recognize the developmental origins of violence and psychopathology (Day et al., 2021; Levy et al., 2015). Adults’ attachment styles may also provide potentially important information to clinicians about clients’ vulnerabilities within the therapeutic relationship.

In summary, our findings support attachment theory’s value in helping to understand the cycle of violence. We showed that maltreated children continued to be at higher risk of violent arrests in middle adulthood, which was partly mediated by greater attachment insecurity in close adult relationships. The potential importance of attachment anxiety in particular emerged, however links between maltreatment subtypes, adult attachment dimensions, and violence require further research. Future work should replicate our findings and test more complex models that help explain individual differences in adult attachment (in)security following maltreatment and how it converges with other mechanisms to influence violence propensity.

## Figures and Tables

**Table 1.** Characteristics of the sample over initial three phases of study

	Records^a^	Interviews			
		1	2
**Dates**	1967–1971	1989–1995	2000–2002
***N***	1575	1196	896
**Characteristics**			
Sex (% male)	49.3	51.3	49.0
White (%)	66.2	62.9	62.2
Black (%)	32.6	34.9	35.2
Hispanic (%)	0.3	3.8	4.0
Other race/ethnicity (%)	1.2	2.2	2.6
Abuse/neglect (%)	57.7	56.5	55.8
Neglect (%)	44.3	45.4	45.3
Physical abuse (%)	10.2	9.2	8.8
Sexual abuse (%)	9.7	8.0	7.6
Mean age at petition (SD)	6.4 (3.3)	6.3 (3.3)	6.2 (3.3)

*Note. SD,* standard deviation.

a ‘Records’ refers to first phase of study, where official records from juvenile (family) and adult criminal court records were used to identify cases of abused and neglected children.

**Table 2.** Descriptive characteristics of the sample (*N* = 892)

Variables	Control, *N* = 395	Any maltreatment, *N* = 497	Neglect, *N* = 404	Physical abuse, *N* = 77
**Demographics**				
Sex, *n* (%)				
Female	193 (48.9)	264 (53.1)	203 (50.2)	38 (49.4)
Male	202 (51.1)	233 (46.9)	201 (49.8)	39 (50.6)
Race, *n* (%)				
White, non-Hispanic	241 (61.0)	302 (60.8)	245 (60.6)	57 (74.0)
Black	146 (37.0)	169 (34.0)	139 (34.4)	15 (19.5)
Other^a^	8 (2.0)	26 (5.2)	20 (5.0)	5 (6.5)
Age at interview 2, *M* (*SD*)	39.59 (3.49)	39.46 (3.56)	39.23 (3.60)	39.78 (3.83)
**Adult attachment, *M* (*SD*)**				
Avoidance	2.63 (0.71)	2.85 (0.74)	2.85 (0.74)	2.84 (0.82)
Anxiety	1.98 (0.91)	2.30 (1.02)	2.28 (1.02)	2.45 (1.04)
**Violent arrests, *n* (%)**				
Arrested after 2000	30 (7.6)	64 (12.9)	53 (13.1)	12 (15.6)

*Note. M*, mean; *SD,* standard deviation.

a Includes Hispanic, Native American, and other racial/ethnic groups, which are combined here to maintain participant confidentiality.

**Table 3.** Correlational matrix showing zero-order Pearson correlations between study variables

Variables	1	2	3	4	5	6	7	8
1. Maltreatment (controls)	–							
2. Neglect (controls)	–	–						
3. Physical abuse (controls)	–	–	–					
4. Female (male)	0.04	0.01	0.004	–				
5. White, non-Hispanic (all other groups)	−0.003	−0.004	0.10*	−0.06	–			
6. Age at interview two	−0.02	−0.05	0.02	0.02	−0.06	–		
7. Attachment avoidance	0.15***	0.16***	0.11*	0.05	−0.08*	0.002	–	
8. Attachment anxiety	0.16***	0.16***	0.18***	0.02	−0.004	−0.004	0.49***	–
9. Violent arrest after 2000 (no violent arrest after 2000)	0.09**	0.09**	0.10*	−0.21***	−0.11***	0.004	0.07*	0.10**

*Note.* Total *N* = 892 (child maltreatment = 497; controls = 395; neglect = 404; physical abuse = 77). Attachment variables are observed mean item response scores for the avoidance and anxiety scales of the Relationship Scales Questionnaire using Simpson et al.’s (1992) operationalization. Categories in parentheses represent the reference group.

* *p* < .05

** *p* < .01

*** *p* < .001.

**Table 4.** Structural equation models predicting paths from childhood neglect and physical abuse to arrests for violence after 2000, through adult attachment anxiety

Adult Attachment Anxiety Model	β (*SE*)	95% CI
*Direct paths*		
Childhood neglect → Attachment anxiety	0.14 (0.04)***	0.07, 0.22
Childhood neglect → Violent arrest after 2000	0.09 (0.06)	−0.02, 0.20
Childhood physical abuse → Attachment anxiety	0.11 (0.04)**	0.04, 0.18
Childhood physical abuse → Violent arrest after 2000	0.09 (0.05)	−0.002, 0.18
Attachment anxiety → Violent arrest after 2000	0.15 (0.06)**	0.04, 0.26
*Indirect paths*		
Childhood neglect → Attachment anxiety → Violent arrest after 2000	0.02 (0.01)*	0.002, 0.04
Childhood physical abuse → Attachment anxiety → Violent arrest after 2000	0.02 (0.01)*	<0.001, 0.03
*R*^2^/m*odel fit*		
*R*^2^ for attachment anxiety	0.03*	
*R*^2^ for violent arrest after 2000	0.21***	
CFI/TLI	0.89/0.83	
RMSEA	0.05	

*Note.* β, standardized regression coefficient indicating change in z-score per unit change in independent variables; *SE* = standard error; CI = confidence interval; CFI = comparative fit index; TLI = Tucker–Lewis index; RMSEA = root mean square error of approximation. All analyses controlled for age, sex, and race.

* *p* < .05

** *p* < .01

*** *p* < .001.

**Table 5.** Structural equation models predicting paths from childhood neglect and physical abuse to arrests for violence after 2000, through adult attachment avoidance

Adult Attachment Avoidance Model	β (*SE*)	95% CI
*Direct paths*		
Childhood neglect → Attachment avoidance	0.15 (0.04)***	0.08, 0.23
Childhood neglect → Violent arrest after 2000	0.10 (0.06)	−0.01, 0.21
Childhood physical abuse → Attachment avoidance	0.06 (0.04)	−0.01, 0.14
Childhood physical abuse → Violent arrest after 2000	0.10 (0.05)*	0.01, 0.19
Attachment avoidance → Violent arrest after 2000	0.10 (0.06)	−0.01, 0.21
*Indirect paths*		
Childhood neglect → Attachment avoidance → Violent arrest after 2000	0.02 (0.01)	−0.003, 0.03
Childhood physical abuse → Attachment avoidance → Violent arrest after 2000	0.01 (0.01)	−0.004, 0.02
*R*^2^/*model fit*		
*R*^2^ for attachment avoidance	0.03*	
*R*^2^ for violent arrest after 2000	0.20***	
CFI/TLI	0.86/0.80	
RMSEA	0.05	

*Note.* β, standardized regression coefficient indicating change in z-score per unit change in independent variables; *SE* = standard error; CI = confidence interval; CFI = comparative fit index; TLI = Tucker–Lewis index; RMSEA = root mean square error of approximation. All analyses controlled for age, sex, and race.

* *p* < .05

** *p* < .01

*** *p* < .001.
